# Photo-Crosslinked Keratin/Chitosan Membranes as Potential Wound Dressing Materials

**DOI:** 10.3390/polym10090987

**Published:** 2018-09-04

**Authors:** Che-Wei Lin, Yi-Kai Chen, Min Lu, Kuo-Long Lou, Jiashing Yu

**Affiliations:** 1 Institute of Biotechnology, National Taiwan University, Taipei 10617, Taiwan; d01642002@ntu.edu.tw; 2 Department of Chemical Engineering, National Taiwan University, Taipei 10617, Taiwan; sleepykai72@gmail.com (Y.-K.C.); b02504041@ntu.edu.tw (M.L.)

**Keywords:** keratin, chitosan, composite membrane, UV-crosslink

## Abstract

In this study, we combined two kinds of natural polymers, chitosan and keratin, to develop a portable composite membrane via UV irradiation. UV-crosslinking without an additional chemical agent makes the fabrication more ideal by reducing reactants and avoiding residual toxic chemicals. This novel composite could perform synergistic functions benefitting from chitosan and keratin; including a strong mechanical strength, biodegradability, biocompatibility, better cell adhesion, and proliferation characteristics. Furthermore, compared with our previous research, this keratin-chitosan composite membrane was improved in that it was made to be portable, enabling it to be versatile and have various applications in vitro and in vivo. Based on these facts, this innovative composite membrane has high potential for serving as an outstanding candidate for wound healing or other biomedical applications.

## 1. Introduction

Natural biocompatible polymers are considered one of the most effective solutions for biocompatibility problems caused by applying biomaterials in vivo [1,2]. There is a current surge in research on biocompatible materials obtained from natural materials including collagen [3,4,5], gelatin [6,7,8], chitosan [9,10], alginate [11,12], keratin [13,14,15], etc. These materials have been utilized to fabricate related products, and their material properties and biocompatibility in the field of tissue engineering have also been investigated.

To develop ideal biomaterials, the materials or polymers chosen should have multiple functions, rather than a single trait that might limit their biomedical application [16]. Based on previous studies, keratin seems to be one of the natural materials with multiple favorable characteristics which could serve as the component of superior biomaterials [17,18]. Keratins, which are cysteine-rich fibrous proteins, can be found in various natural filamentous or hard structures such as hairs, wools, nails, etc., with characteristics of bioactivity, biocompatibility, biodegradability, and natural abundance [19,20]. Keratin has been widely used in biomaterials especially for hemostasis, wound healing, and peripheral nerves repair [21,22,23]. A previous study indicated that keratins extracted from human hair fibers contain a cell adhesion motif of leucine-aspartic acid-valine (LDV) [24,25]. Hence, research on keratin coating or biomaterials for in vitro cell culture through investigating the attachment and growth of different cell types has been developed [26,27], and the results showed better growth on keratin than standard controls, such as tissue culture plastic or other biomaterials such as collagen [28].

However, the main disadvantage of keratin was shown to be its poor mechanical properties. Thus, there have been many studies conducted to improve the mechanical properties of products by modification and crosslinking with other materials [29,30,31]. This approach could not only make keratin more versatile, but also produce synergistic effects if the materials can be suitably prepared. On the other hand, chitosan has been widely investigated as a natural biomaterial for many applications not only due to its biocompatibility, biodegradability, and antimicrobial properties [32,33], but also its effective mechanical stability and good film-forming capacity, which makes it useful for the development of chitosan-based films [34,35,36]. Previous studies also demonstrated that the UV-crosslinked chitosan conjugated with Arginyl-glycyl-aspartic acid (RGD) enhanced the mechanical strength simultaneously and maintained cell attachment [37,38]. Thus, we were interested in combining chitosan and keratin by the UV-crosslinking method. Although there are some reports indicating the use of acetic acid as a crosslinking agent for keratin-chitosan composite film [39], to the best of our knowledge, there are very few studies on keratin materials crosslinked via UV irradiation. Through this method, we aimed to avoid the disadvantages resulting from chemical crosslinking such as the residual chemical agent and the time-consuming crosslinking procedure.

Our previous research reported that the stability, mechanical strength, and biocompatibility of a keratin-chitosan coating dish could be improved [40], and we also analyzed the coating dish for bio-suitability by seeding human adipose stem cells (hASCs) onto them in vitro. Based on previous experience, we fabricated a new portable keratin-chitosan composite membrane by UV induction, and made it portable so that it could be applied to various fields. The objective of this study is to verify the feasibility and superiority of the keratin-chitosan composite membrane in a portable form. The evaluations were determined via fourier-transform infrared spectroscopy (FTIR), scanning electron microscopy (SEM), contact angle, and fluid absorption experiments. Afterwards, cell morphology, cell viability, proliferation, and migration ability were evaluated. Finally, an in vivo subcutaneous implantation experiment was conducted to verify whether the composite membrane would have a favorable biocompatibility. This innovative keratin-chitosan composite membrane could be employed in wound dressing and other biomedical applications.

## 2. Materials and Methods

### 2.1. Extraction of Human Hair Keratins

Pure Keratin solution was prepared from human hair according to the method reported previously [41]. In brief, human hair (10 g) was cut and washed with distilled water, and then steeped in a 600 mL mixed liquor of methanol/chloroform (2:1, *v*/*v*) (ECHO Chemical Co., Miaoli, Taiwan) for 18–24 h to remove lipid covering the external surface of human hair. Afterwards, the hair was air-dried and soaked in an extraction solution (5 M urea, 25 mM Tris–HCl, 5% 2-mercaptoethanol (β-ME) and 2.6 M thiourea) at 50 °C for 72 h. The extracted solution was filtered through a 0.22 µm filter (Thermo, Waltham, MA, USA) and centrifuged at 5000 *g* for 30 min at 4 °C. The supernatant was exhaustively dialyzed against the water (pH 10.5) and the water was refreshed seven times in 24 h at 4 °C. Finally, the protein concentration of keratin was then determined using Protein Assay Dye Reagent (Bio-Rad, Hercules, CA, USA).

### 2.2. Chitosan-Azide Synthesis

The synthesis of chitosan-azide (CHI-AZ) was reported by the protocol of previous research [40,42]. In brief, 80 mg 4-azidobenzoic acid (ABA) (TCI, Tokyo, Japan), 47.75 mg N-(3-dimethylaminopropyl)-N0-ethylcarbodiimide hydrochloride (EDC) (Sigma-Aldrich, St. Louis, MO, USA), and 23 mg *N*-Hydroxysuccinimide (NHS) (Sigma-Aldrich, St. Louis, MO, USA) were prepared. Afterwards, 400 mg chitosan (Mw of 50–90 kDa with DD of 80%, Sigma-Aldrich, St. Louis, MO, USA) was added to 30 mL mixed solution (water/DMSO (Sigma-Aldrich, St. Louis, MO, USA) (1:1, *v*/*v*)), and then previously prepared chemicals were added to the solution. The pH value was adjusted to 5 using 1 M HCl, and the reactants were stirred overnight at room temperature in the dark. After stirring overnight, the solution was centrifuged at 4500 rpm for 1.5 h, and the supernatant was taken out. The supernatant solution was exhaustively dialyzed against deionized water through a seamless cellulose tube (MWCO: 12,000–14,000, Cellu Sep, Seguin, TX, USA) to remove unreacted reactants, and the solution was then lyophilized for two days to obtain our final product.

### 2.3. Fabrication of Keratin-Chitosan Membranes

A previously developed UV-crosslink protocol from another study was applied to the fabrication of the keratin-chitosan coating dish [40]. In this study, we established different mixing ratios of keratin and chitosan-azide solution, as shown in Table 1. The keratin-chitosan solutions were poured into an O-ring mold (150 μL each), and were then exposed to UV radiation for 15 min (UniVex, UT-500UV, 100 mW cm^2^ at 365 nm), which were subsequently air-dried in the dark for 16–24 h. The obtained membranes were thereafter designated as KECHI/0.25:1, KECHI/0.5:1 and KECHI/1:1 composite membranes and stored in a desiccator for the following experiments.

### 2.4. Fourier Transform Infrared (FTIR) Spectra Analysis

Attenuated Total Reflection (ATR)-FTIR type spectra were obtained using a Nicolet NEXUS 470 spectrometer (Thermo, Waltham, MA, USA). The spectrum scope of samples was taken between 4000 cm^−1^ and 650 cm^−1^.

### 2.5. SEM Images

The surface morphology of the keratin–chitosan membranes was observed by scanning electron microscopy (SEM). For SEM pre-treatments, the membranes were freeze-dried for 18–24 h, coated with gold by sputter deposition, and then observed at 300× magnification under a scanning electron microscope (NovaTM NanoSEM 230, Thermo, Waltham, MA, USA). Images were then processed and analyzed via Image J software.

### 2.6. Fluid Absorption

The fluid absorption ability of the keratin-chitosan membranes was measured by soaking dry membranes in PBS solution (Sigma, St. Louis, MO, USA) at 25 °C for different time durations including 0 min, 30 min, 60 min, 90 min, 120 min, and 150 min. In each period, the keratin-chitosan membranes were taken from solution, the excess PBS was removed with filter paper, and the membranes were weighted.

The fluid uptake percentage (W%) of these keratin-chitosan membranes was calculated using the formula:

W% = (W wet − W dry)/W dry × 100%

### 2.7. Contact Angle

A contact angle goniometer (FTA125, NIST, Gaithersburg, MD, USA) was used for the water contact angle measurements at room temperature. The volume of the water droplet was fixed at 5.0 μL, and the contact angle was determined 10 s after the water droplet was deposited on the surface of the keratin-chitosan composite membranes. A plugin for the MagicDroplet software was exploited to estimate the contact angle values, and the average value of ten measurements performed at different surface locations was reported as the contact angle of the composite membranes.

### 2.8. Cell Culture of L929

Cells selected for the present experiment were a cell line of mouse fibroblasts—L929, and the cells were cultivated in a 75T flask and Dulbecco’s modified eagle medium (DMEM, Sigma, St. Louis, MO, USA) supplemented with 10% FBS (Sigma, St. Louis, MO, USA), and 1% antibiotics solution (Sigma, St. Louis, MO, USA). Cells were incubated at 37 °C in an incubator containing 5% CO_2_.

### 2.9. Cell Seeding on Keratin–Chitosan Membranes

The keratin–chitosan membranes were used to evaluate cytocompatibility. All the membranes were rinsed with PBS solution. Finally, the membranes were immersed in culture medium (DMEM) at room temperature in 48 well culture plates for 30 min before L929 (5 × 10^5^/well) was seeded onto the composite membranes. After being incubated for 4 h in a CO_2_ incubator at 37 °C, the membranes were supplemented with 1.5 mL complete medium. The culture medium was changed every two days.

### 2.10. Water Soluble Tetrazolium-1(WST-1) Assay

The cell viability of L929 cells on the membranes was observed by the WST-1 assay (CytoScan™ WST-1 Cell Proliferation Assay, G-Biosciences, St. Louis, MO, USA). WST-1 Tetrazolium Salt was used to measure the viability of L929 cells (5 × 10^5^/sample membrane) being cultivated on the keratin–chitosan membranes at 37 °C every day until day 5. At the time point for the cell viability assay, the membranes were incubated in 0.5 mL DMEM medium without phenol red containing 50 μL, 5 mg/mL WST-1 solution at 37 °C for 1 h. The samples were shaken for 1 min, and the absorbance was measured by using a microplate reader at 420–480 nm and setting the reference wavelength to more than 600 nm.

### 2.11. Immunofluorescence Staining

Phalloidin is a selective bicyclic peptide that is used for staining F-actin. It is observed for all variants of an F-actin shape in many different species of cell. After five days of cultivation, the keratin-chitosan membranes with cells were rinsed with PBS and subjected to immunofluorescence staining. After fixing with ice-cold methanol/acetone for 5 min, the sample were permeabilized with 0.1% Triton-X100 for 5 min, and then incubated with phalloidin antibody (1:200, Abcam, Cambridge, UK) at room temperature for 20–90 min. Complexes were incubated with Hoechst 33342 (Thermo Fisher Scientific, Waltham, MA, USA) for nuclei staining. Finally, the sample membranes were observed and examined by using the Zeiss LSM 780 confocal microscope. Images were then processed and analyzed via Image J software (NIH, Bethesda, MD, USA).

### 2.12. Cell Migration Assay

The cell migration assays were carried out on keratin-chitosan membranes using the Culture-Insert well (Thistle Scientific, Uddingston Glasgow, UK). When the Culture-Insert well is placed on a keratin-chitosan membrane, it provides two cell culture reservoirs which are separated by a thick wall.

The L929 cells (5 × 10^5^/well) are cultured in both reservoirs and the silicone insert is then removed from the surface. This move results in two precisely defined cell patches, which are separated by a zone that is exactly the same width as the separation wall. After barrier removal, the membranes were washed three times with PBS to remove dead cells and then fresh growth medium was added. The cells were incubated over the patterned areas for up to 16 h and were used to evaluate the migration ability of L929 cells on keratin–chitosan membranes by using an optical microscope at 0 h, 2 h, 6 h, 8 h, and 16 h. Finally, the Image analysis was done via the Image J software.

### 2.13. Evaluation of In Vivo Biocompatibility of Keratin-Chitosan Membrane in a Mouse Model

In brief, the subcutaneous implantation of keratin-chitosan membranes was performed in ICR (CD1) mice (25.2 ± 0.7–27.4 ± 0.5 g, seven weeks, male), and the experimental mice were divided into different experimental groups (six mice for each group) and used to evaluate the biocompatibility of keratin-chitosan membranes in subcutaneous tissue for seven days. The experiments were under constant monitoring, and followed the guidelines (Animals and Implantation) of the National Taiwan University Animal Center standard. At day 7, subcutaneous tissue of each group of mice, including the surrounding implantation of membranes of margin skin, were obtained after the mice were euthanatized. Finally, the samples were embedded in Optimal cutting temperature compound (OCT compound), sectioned, and stained with hematoxylin and eosin (H&E) for observations.

### 2.14. Statistical Analysis

The data were analyzed via the ANOVA test and by using GraphPad Prism 7 software (GraphPad software, La Jolla, CA, USA). Data were presented as the mean ± SD (standard deviation). A value of *p* ≤ 0.05 was considered statistically significant.

## 3. Results and Discussion

### 3.1. Characterization of Keratin-Chitosan Membranes

**Figure 1:**
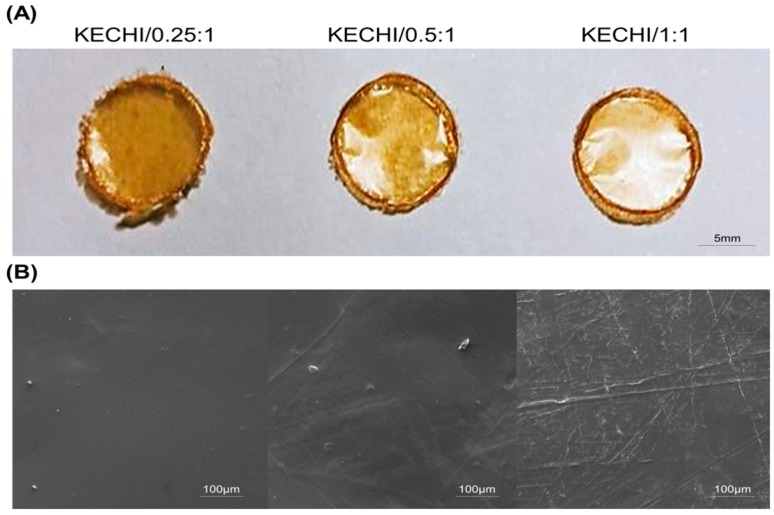
(**A**) Appearance of Keratin-chitosan membranes; (**B**) SEM images of membrane (KECHI/0.25:1, KECHI/0.5:1, and KECHI/1:1).

Keratin-chitosan composite membranes showing a semitransparent appearance after being dried and removed from the O-ring are shown in Figure 1A. After blending chitosan with keratin, macroscopically uniform, yellowish, optically semitransparent membranes were obtained. The surfaces of the keratin-chitosan composite membranes look rather smooth, without obvious cracks or holes. Besides, the appearance of these membranes did not change, even when different keratin/chitosan ratios were used, and all membranes could be easily removed from the O-ring mold.

The surface morphology of the keratin-chitosan composite membranes was examined by SEM (Figure 1B). The SEM images reveal that the samples (KECHI/0.25:1, KECHI/0.5:1, and KECHI/1:1) had a compact and continuous structure, and voids were not found, even at higher magnifications, showing that membranes had great structural integrity. Nevertheless, compared with the KECHI/0.25:1 group, the surfaces of KECHI/0.5:1 and KECHI/1:1 groups were less smooth, with some pleated bulges appearing at higher magnifications, which can probably be attributed to the different concentrations of keratin.

**Figure 2:**
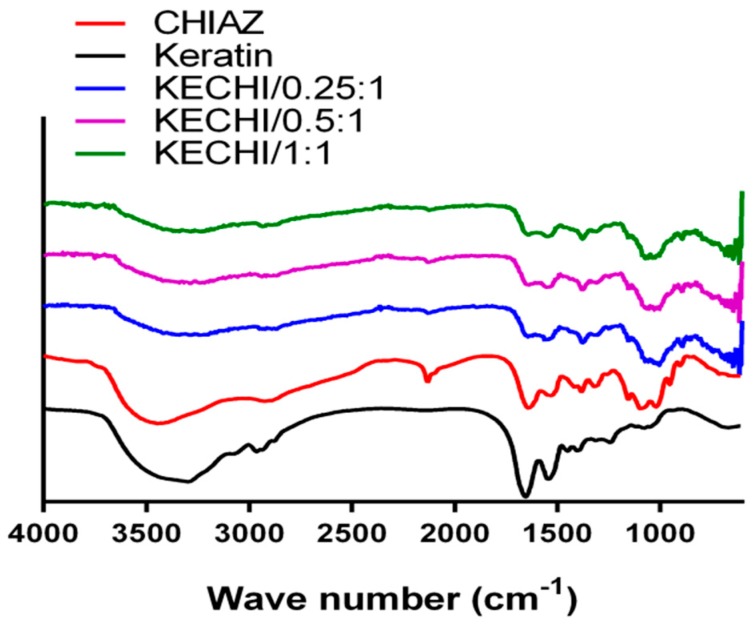
The FTIR results of keratin-chitosan membranes: Black: KECHI/0.25:1; Red: KECHI/0.5:1; Blue: KECHI/1:1.

FTIR analysis was used to verify the interactions between chitosan and keratin in the composite membranes (Figure 2). A peak at 2118 cm^−1^ indicated the graft of the azide functional group on chitosan (2110–2160 cm^−1^). When exposing the mixture of KE/CHI-AZ to UV light, this characteristic peak disappeared, indicating successful crosslinking between chitosan and keratin. The ether characteristic band between 1058 and 1320 cm^−1^ was a signature of chitosan. On the one hand, the major bands related to keratin are observed, the band at 1641 cm^−1^ is assigned to the C=O group, and the peak at 1551 cm^−1^ is attributed to in plane bending of NH group, while the signal at 1394 cm^−1^ is related to bending of the CH_3_ groups [43]. Meanwhile, a clear shoulder is found at 1029 cm^−1^ in the spectra of short and large fibers of keratin.

**Figure 3:**
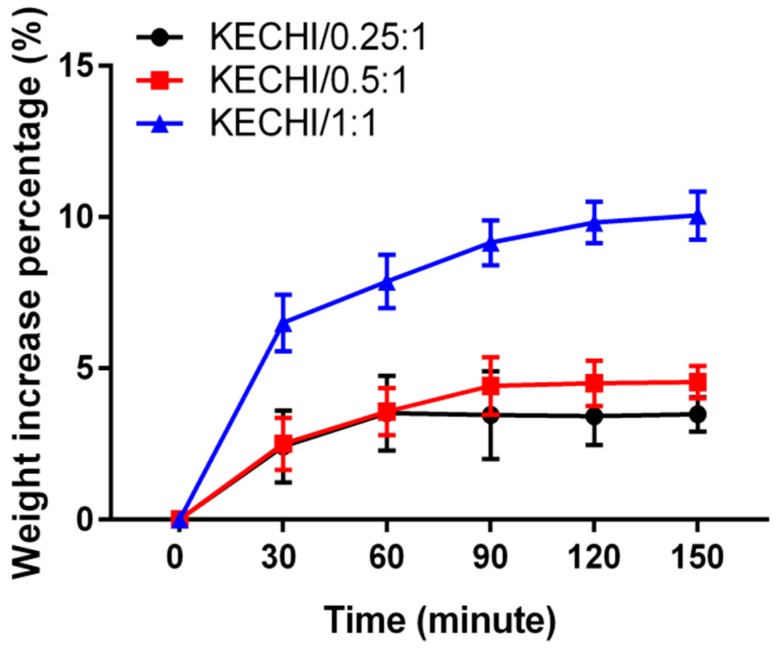
Fluid absorption of keratin-chitosan membranes immersed in pH 7.4 PBS solution.

To verify the fluid absorption ability of different compositions of keratin-chitosan composite membranes, we immersed three kinds of membrane into pH 7.4 PBS solution (Figure 3). The result shows that KECHI/0.25:1 and KECHI/0.5:1 groups only increased by less than 5% of weight by absorbing fluid and rapidly reached equilibrium after being soaked for 90 min. In contrast, KECHI/1:1 increased by 10% of weight, which is approximately a two-fold increase that resulted from the better hydrophilicity provided by the increased concentration of keratin in the keratin-chitosan composite membrane.

**Figure 4:**
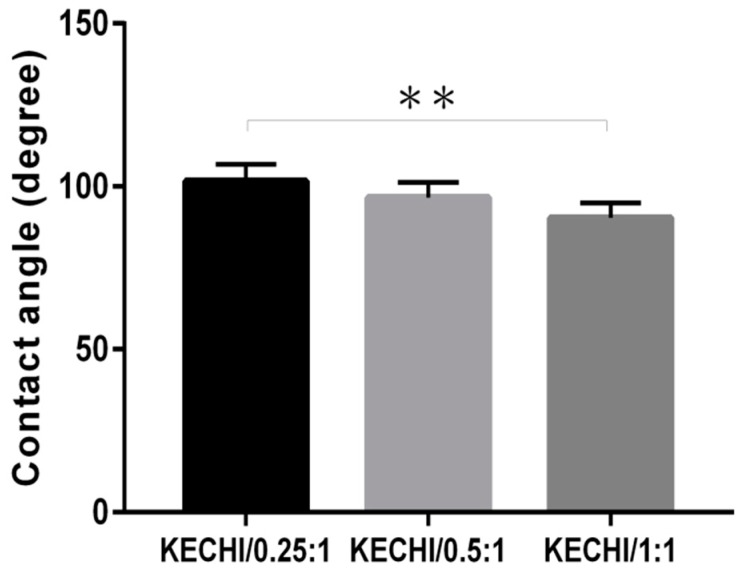
Quantification of contact angle of KECHI/0.25, KECHI/0.5, and KECHI/1:1. ** *p* < 0.01.

The water contact angles of keratin/chitosan membranes were measured as a characteristic of different keratin concentrations (Figure 4). Compared with KECHI/0.25:1 (101 ± 3°), the water contact angles slightly decreased to 95° in KECHI/0.5:1 (95 ± 4°), when the keratin concentration was higher (KECHI/1:1), and the water contact angles decreased to 86° (86 ± 3°). The contact angles of all the samples increased in the order of KECHI/0.25:1 > KECHI/0.5:1 > KECHI/1:1. Thus, the water contact angles decreased with the increase of keratin concentration, indicating that the KECHI/1:1 was more hydrophilic than KECHI/0.25:1 and KECHI/0.5:1 groups. The possible reason for this might be ascribed to the better hydrophilicity, the morphological variation, and the crosslinking degree of the membranes due to the different keratin concentration ratios.

**Figure 5:**
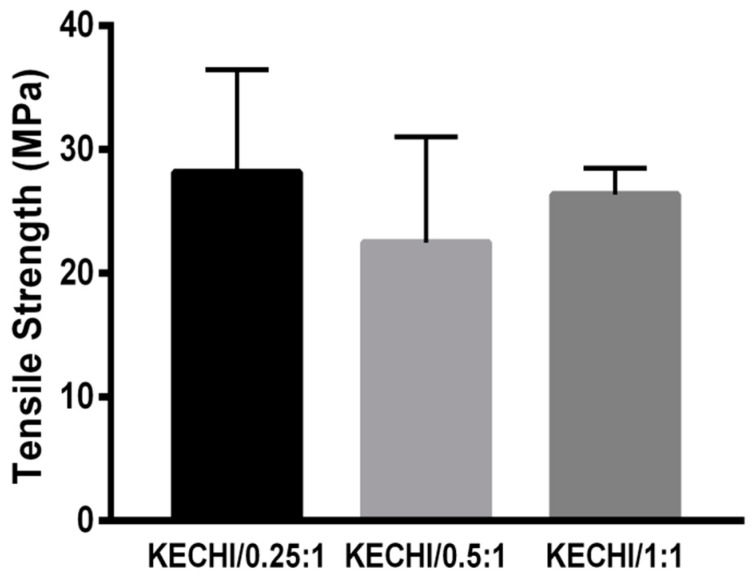
Tensile strength of keratin-chitosan composite membranes.

The strong tensile strength of biomaterials to resist external force plays a significant role in biomedical applications such as scaffolding, wound dressing, etc. As Figure 5 shows, different compositions of membrane (KECHI/0.25:1 (28.13 ± 8.31 MPa), KECHI/0.5:1 (22.47 ± 8.55 MPa), and KECHI/1:1 (26.33 ± 2.14 MPa)) had a similar tensile strength. This fact points out that the mechanical properties of membranes are mostly influenced by CHIAZ, which was designed at the same concentration in every composite membrane. The strong mechanical strength was mainly provided by the natural property of chitosan and crosslinking degree of azide functional groups. Therefore, it is reasonable to have a similar tensile strength if the same concentration of CHIAZ is utilized when fabricating these membranes.

### 3.2. Cellular Viability on Keratin-Chitosan Membranes

Quantitative analysis of cell attachment, viability, and proliferation on biomaterials is essential. To analyze the biocompatibility of keratin/chitosan membranes, an immunofluorescence stain, WST-1 assay, and double-stranded DNA (dsDNA) content were carried out in all KECHI/0.25:1, KECHI/0.5:1, and KECHI/1:1 groups.

**Figure 6:**
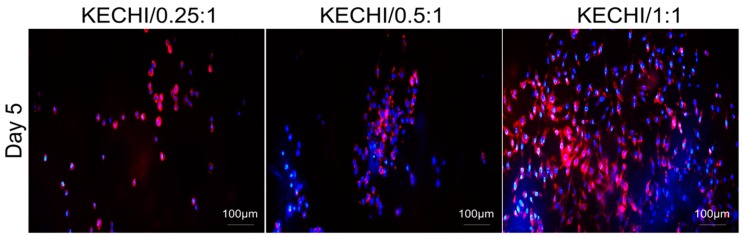
Immunofluorescence staining of L929 cells seeded on keratin-chitosan membranes. Confocal microscopy images of L929 stained with phalloidin for F-actin (red), and nuclei counterstained with DAPI (blue) in all keratin-chitosan membranes at day 5.

The cytoskeleton plays important roles in cell morphology, adhesion, and gene expression. To detect the influence of the keratin-chitosan membrane on the cytoskeletal architecture of the L929 cells, the F-actin cytoskeleton was examined after five days of cell incubation. Phalloidin staining observations revealed that the L929 cells attached evenly and had well stretched pseudopodia on the KECHI/1:1, whereas the majority of cells on the KECHI/0.25 and KECHI/0.5:1 composite membranes were localized and spherical in shape (Figure 6).

**Figure 7:**
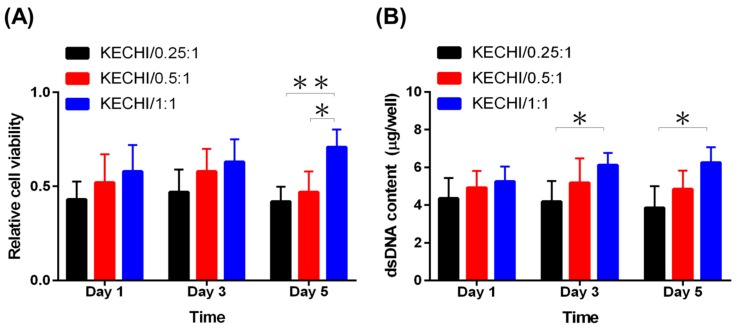
Viability of L929 cells on the keratin/chitosan membrane at days 1, 3, and 5 of cell culture. (**A**) WST-1 assay of L929 cells on KECHI/0.25:1, KECHI/0.5:1, and KECHI/1:1 membranes. (**B**) Proliferation of L929 cells in contact with keratin/chitosan membranes by measuring dsDNA content. * *p* < 0.05, ** *p* < 0.01.

The WST-1 assay, a colorimetric assay, is employed for determining the cell viability, involving the reduction of WST-1 reagent, giving an amber color formazan product in the presence of viable cells. Hence, the absorbance of formazan product proportionally reflects the existence of viable cells. At day 1 and 3, cells cultured on KECHI/1:1 displayed a slightly higher viability than those on KECHI/0.25 and KECHI/0.25, but there was no statistical difference. At day 5, the KECHI/1:1 group had the highest cell viability level, while it was higher on KECHI/0.5:1 than on KECHI/0.25:1(Figure 7A). At the same time, the cell number estimated by the dsDNA assay demonstrated L929 proliferation on KECHI/0.25:1, KECHI/0.5:1, and KECHI/1:1 composite membranes. After three days of culture, the dsDNA content of the L929-seeded KECHI/1:1 keratin-chitosan composite membrane slightly increased (Figure 7B).

However, on day 5 of culture, the dsDNA content of the KECHI/1:1 group cultured on L929 was significantly higher than the KECHI/0.25:1 group (6.2 ± 0.8 μg vs. 3.8 ± 0.7 μg per well at day 5, *p* < 0.05) and KECHI/0.5:1 group (6.2 ± 0.8 μg vs. 4.8 ± 0.8 μg per well at day 5; Figure 7B).

In contrast to the KECHI/1:1 group, the L929-seeded KECHI/0.25:1 and KECHI/0.5:1 groups gradually decreased in dsDNA content throughout the culture period [KECHI/0.25:1 group (4.2 ± 0.8 μg at day 1; 3.8 ± 0.7 μg at day 3); KECHI/0.5:1 group (5.2 ± 0.9 μg at day 3; 4.8 ± 0.9 μg at day 5)].

This finding suggested that cell viability for the low concentration of keratin-chitosan composite membranes was quite limited, and the KECHI/0.25:1 and KECHI/0.5:1 group were indeed confirmed to demonstrate a low cell viability by the WST-1 assay and dsDNA content assay. A previous study showed that the properties of the UV-crosslinked keratin/chitosan coating dish could be greatly affected by different keratin concentrations [40]. We also found that an inadequate keratin concentration in KECHI/0.25:1 and KECHI/0.5:1 groups resulted in poor cell attachment, demonstrated by the paucity of cells and the round cell shape on the membranes’ surface three days after seeding cells. Therefore, the analysis of phalloidin staining, WST-1 assay, and dsDNA content revealed a virtually higher cell viability in the KECHI/1:1 group throughout the culture period.

### 3.3. Cell Migration on Keratin-Chitosan Membranes

**Figure 8:**
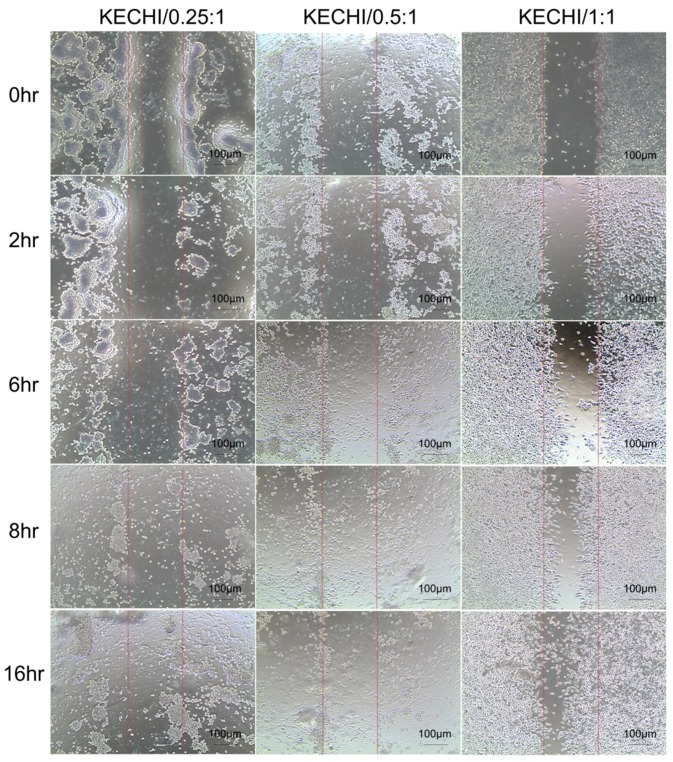
Effects of different types of keratin-chitosan membranes on cell migration of L929 cells. After removing the Culture-Insert well mold, the cell-free zones were photographed at 0 h, 2 h, 6 h, 8 h, and 16 h.

We wanted to demonstrate the feasibility of keratin-chitosan composite membranes for cells migrating from confined patterns at a desired time point. We conducted a simple experiment, subject to an in vitro wound healing assay, by using a Culture-Insert mold, and L929 cells were then seeded in a Culture-Insert mold area on composite membranes and cell migration ability was observed using a optical microscope. Afterwards, we observed the images of cells at the area of the composite membranes and the scratched wound area at 0 h, 2 h, 6 h, 8 h, and 16 h. The results of cells’ migration by the scratch wound migration assay are shown in Figure 8. It can be seen from the image that the KECHI/1:1 group showed the largest migration distance at almost every duration when compared with the other groups. Image observation revealed that the cell migration distance on the KECHI/1:1 group was significantly longer than that on KECHI/0.25:1 and KECHI/0.5:1 at 6 h, 8 h, and 16 h. We also found that more L929 cells migrated from the Culture-Insert area into the scratched wound area as the keratin content in the composite membranes increased (Figure 8). Compared to the 1:1 group, only KECHI/0.25:1 and KECHI/0.5:1 groups exhibited significantly lower cell numbers in the scratched wound area. In our previous study, we showed that a composite keratin/chitosan coating dish supports initial hASC attachment, followed by the proliferation ability of cells from the coating dish at a later stage [44]. This conclusion was also proved indirectly via the cell migration ability of a high concentration of keratin in composite membranes above. Overall, the results of the distance of migrated cells across the wound repair duration illustrated that a higher concentration of keratin could enhance cell migration.

### 3.4. Subcutaneous Implantation of Keratin-Chitosan Membranes

**Figure 9:**
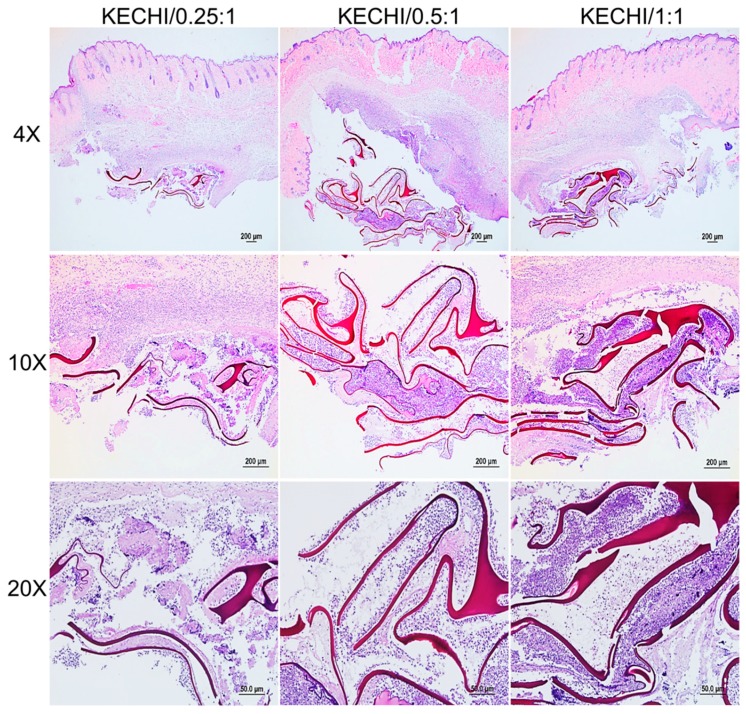
Histological sections of keratin-chitosan membranes subcutaneously implanted after one week.

The histological sections of keratin-chitosan membranes implanted subcutaneously are shown in Figure 9 to acquire the initiatory data about the biodegradability and biocompatibility of keratin-chitosan membranes. It was generally believed that acute inflammation is the direct response of a tissue to implantation at day 7 [45,46]. The inflammation response was mainly located at the outer surface region of the membranes, tissue fluid, and emigration of immunocytes from blood vessels to the area of implantation [47,48]. In a 10× histological section of the subcutaneous tissue, we also observed that the severe deterioration of the overall membranes’ architecture is evident, and the obvious inflammatory cells could be observed in its inner region. However, compared with the architectural integrity before the implantation of membranes, the obvious membranes’ deformation could be observed in the implantation region. It is worth noting that all keratin-chitosan membranes were broken at the same state of Implant conditions.

This result is similar to a previous experiment of the mechanical property test. In the 20× histological section, the keratin residues were mainly located at the bottom of the subcutaneous tissue and densely populated with cells (Figure 9). According to the previous research, a broad range of hydrolytic enzymes, like lysozyme and cellulase, are found to be the primary enzymes responsible for chitosan degradation in mammals [49]. Additionally, the in vivo biodegradation of biomaterials is also dependent upon the material structure, the implantation site, and function [32,50]. Overall, the subcutaneous implantation of keratin-chitosan membranes indicated a very fast biodegradation, and its biodegradation rate in the wound may be related to a repair-triggering cascade of robust cellular activities, together with the presence of a higher number of enzymes [51].

## 4. Conclusions

In material evaluation, the result verified that this composite membrane had an integrated appearance, moderate fluid absorption, and strong tensile strength. With these merits, this material qualified for further in vitro and in vivo experiments. The result of the cellular response in vitro demonstrated that this membrane could have better cell adhesion, proliferation, and migration if a higher concentration of keratin was utilized. Moreover, the biocompatibility and biodegradability could be proved by the mild inflammation and degradation of the membrane observed in the experiment of subcutaneous implantation. All the positive results showed that this portable keratin-chitosan composite membrane could be a desirable biocompatible biomaterial for uses such as wound dressing, and could have a promising future in various biomedical applications.

## Figures and Tables

**Table 1:** Experimental design of keratin and chitosan-azide solution for membrane preparation.

	Keratin (mg mL^−1^)	Chitosan (mg mL^−1^)
KECHI/0.25:1	1.25	5.00
KECHI/0.5:1	2.50	5.00
KECHI/1:1	5.00	5.00
